# Learning strategies and general cognitive ability as predictors of gender- specific academic achievement

**DOI:** 10.3389/fpsyg.2015.01238

**Published:** 2015-08-19

**Authors:** Stephanie Ruffing, F. -Sophie Wach, Frank M. Spinath, Roland Brünken, Julia Karbach

**Affiliations:** ^1^Department of Psychology, Saarland University, SaarbrueckenGermany; ^2^Department of Education, Saarland University, SaarbrueckenGermany; ^3^Department of Psychology, Goethe University Frankfurt, FrankfurtGermany

**Keywords:** academic achievement, learning strategies, gender differences, general cognitive ability, multi-group analyses

## Abstract

Recent research has revealed that learning behavior is associated with academic achievement at the college level, but the impact of specific learning strategies on academic success as well as gender differences therein are still not clear. Therefore, the aim of this study was to investigate gender differences in the incremental contribution of learning strategies over general cognitive ability in the prediction of academic achievement. The relationship between these variables was examined by correlation analyses. A set of *t*-tests was used to test for gender differences in learning strategies, whereas structural equation modeling as well as multi-group analyses were applied to investigate the incremental contribution of learning strategies for male and female students’ academic performance. The sample consisted of 461 students (mean age = 21.2 years, SD = 3.2). Correlation analyses revealed that general cognitive ability as well as the learning strategies effort, attention, and learning environment were positively correlated with academic achievement. Gender differences were found in the reported application of many learning strategies. Importantly, the prediction of achievement in structural equation modeling revealed that only effort explained incremental variance (10%) over general cognitive ability. Results of multi-group analyses showed no gender differences in this prediction model. This finding provides further knowledge regarding gender differences in learning research and the specific role of learning strategies for academic achievement. The incremental assessment of learning strategy use as well as gender-differences in their predictive value contributes to the understanding and improvement of successful academic development.

## Introduction

A great deal of research has focused on the explanation and prediction of academic performance (AP), particularly because it is of high social and individual interest (Spinath, 2012). For instance, AP is highly correlated with social wealth and it also is a strong predictor for vocational career success and socioeconomic prosperity. Research on AP in college students revealed a female advantage in performance and persistence (e.g., Conger and Long, 2010), referred to as the “gender gap” in educational achievement. As a consequence, the search for factors underlying those discrepancies is of great interest in educational psychology.

Important factors in the prediction of AP are traditional cognitive variables, such as college admission tests, on the one hand, and psychosocial or non-cognitive predictors, like motivational variables, on the other hand (Robbins et al., 2004; Credé and Kuncel, 2008). The impact of general cognitive ability, which belongs to the category of cognitive determinants of AP, is well documented throughout previous research (e.g., Kuncel et al., 2004; Rohde and Thompson, 2007) indicating that it is the most powerful single predictor of academic achievement (e.g., Spinath et al., 2006). For instance, general cognitive ability was significantly related with academic grades in school pupils (Spinath et al., 2006) as well as in college students (Richardson et al., 2012). Besides, there is also evidence that general cognitive ability is associated with a various set of different academic and vocational success criteria (Kuncel et al., 2004). The importance of general cognitive ability is not limited to performance criteria (for an overview, see e.g., Brand, 1987), but it is also a construct of major importance for a broad set of life outcomes and behaviors (Kuncel et al., 2004). Consistently, general cognitive ability is probably the most researched trait in psychological research (Gottfredson, 2002). However, general cognitive ability is also considered relatively stable (Gottfredson, 2002) and given that a main interest in education is not only the understanding but also the improvement of achievement processes (Spinath et al., 2006), there is also a high interest in more changeable determinants of AP.

Thus, the main challenge faced by previous research was to improve the prediction of students’ academic success by identifying additional factors explaining incremental variance over general cognitive ability in order to get a more differentiated and improved prediction of AP. Richardson et al. (2012, p. 353) accordingly stated “that predictions of AP may be more accurate if they are based on assessment of a variety of individual differences, not just of past achievement and cognitive capacity.” Credé and Kuncel (2008) also highlighted the importance of incorporating non-cognitive factors to reduce the adverse impact of substantial group differences in cognitive predictors and to increase the accuracy of admission decisions. In addition to prior grades and standardized tests, the aspects these authors considered most important are study habits and skill measures. A further advantage of including learning strategies in the prediction of AP is the fact that they are considered less stable than cognitive ability (Richardson et al., 2012). Accordingly, a better understanding of learning strategies in the educational process may also help students to modify and improve their strategy use. Therefore, it may have further implications for structural issues in the organization of the academic system including, for example, how lecturers structure their courses. This point may also be of special interest in the education of teacher students, because they not only need to apply learning behavior in their own studies, but they also serve as models for their students in their subsequent professional life (Hagger et al., 2008). Although there was some research on the impact of learning behavior on AP (e.g., Diseth and Kobbeltvedt, 2010; Diseth, 2011), research controlling for the powerful predictor general cognitive ability and focusing specific learning strategies is more rare.

A further gap in prior research concerns the examination of gender differences in the predictive value of learning strategies for AP. There are many studies showing individual differences in the application of learning strategies between male and female students (e.g., Schiefele et al., 2003). Therefore, a gender-specific approach in the prediction of AP might be necessary to understand gender differences in AP (e.g., Conger and Long, 2010) and to account for results showing different predictive values for males and females (e.g., Mellon et al., 1980). Consequently, it is essential to determine which learning strategies male and female students use differently and to investigate their relative importance for AP. However, to our knowledge no previous study examined the contribution of learning strategies and general cognitive ability for college students’ AP in one model while considering gender differences.

Therefore, the aim of the present study was to investigate both the cognitive predictor general cognitive ability and the non-cognitive predictor learning strategies in the prediction of AP. Considering evidence regarding gender-specific learning behavior (e.g., Schiefele et al., 2003) and findings of gender differences in the predictability of AP in the literature (Rosander and Bäckström, 2012), we additionally tested for gender differences in the prediction of AP.

Ever since Binet and Simon (1916) reported evidence for a close association between children’s cognitive ability and their performance in school, numerous studies have investigated this relationship. These studies showed that intelligence is a powerful predictor for AP in a number of academic contexts, including college and university (e.g., Kuncel et al., 2004; Rohde and Thompson, 2007). However, selection procedures in tertiary educational settings (e.g., through grades or admission tests) reduce variation in intelligence within admitted samples of students. Thus, a recent meta-analysis reported a correlation of 0.20 between cognitive ability and grade point average (GPA; Richardson et al., 2012). These findings indicated that after controlling for general cognitive ability much of the variance in AP remains unaccounted for. Given that recent literature pointed to the importance of self-regulated learning for AP (for a review, see Zimmerman, 1990), the present study focused on one specific aspect of self-regulated learning, namely the role of learning strategies for individual differences in college students’ AP.

Over the last decades, there has been increasing interest in psychosocial or non-cognitive determinants of AP (e.g., Sackett et al., 2001; Robbins et al., 2004). In the present study, “non-cognitive” refers to “behavioral dispositions, tendencies, and habits that are not measured by typical cognitive tests, such as tests of school performance, ability, and aptitudes.” (Lee and Stankov, 2013, p. 119–120). An important non-cognitive predictor of AP is the construct of study skills; Credé and Kuncel (2008, p. 425) even stated that “overall, study habit and skill measures improve prediction of AP more than any other non-cognitive individual difference variable examined to date”. In contrast to highly structured school environments, studying at a college is less externally regulated and particularly requires the efficient use of self-directed and self-managed learning strategies. Thus, the way students organize learning activities may be an essential predictor of their academic success.

Research on students’ learning behavior increased extensively in the last decades and different research tools, terms, and models have been developed (Cano-Garcia and Justicia-Justicia, 1994; Entwistle and McCune, 2004; Virtanen and Nevgi, 2010). Learning strategies as “behaviors and thoughts that a learner engages in during learning and that are intended to influence the learner’s encoding process” (Weinstein and Mayer, 1986, p. 315) are comprised in all recent theories of strategic and self-regulated learning (Weinstein et al., 2011). Pintrich (1999, p. 459–460) also concluded that “most models assume that an important aspect of self-regulated learning is the students’ use of various cognitive and metacognitive strategies to control and regulate their learning” and described a model of self-regulated learning including three general categories of strategies. This classification includes cognitive (e.g., organization), metacognitive (e.g., planning), and resource-management (e.g., effort management) abilities and can be measured by means of the *Motivated Strategies for Learning Questionnaire* (MSLQ; Pintrich et al., 1991) as well as the German adaption, the *Inventory for the Measurement of Learning Strategies in Academic Studies* (LIST; Wild and Schiefele, 1994).

In a recent meta-analysis by Richardson et al. (2012), the strongest association with AP was established for effort regulation (*r* = 0.32). Nevertheless, other learning strategies, such as metacognition (*r* = 0.18), critical thinking (*r* = 0.15), elaboration (*r* = 0.18), time/study management (*r* = 0.22), and help seeking (*r* = 0.15) showed lower yet significant positive correlations with AP. Griffin et al. (2012) also reported significant correlations between learning scales, such as self-testing, time management, test strategies, study aids, information processing, concentration, attitude, and AP ranging from *r* = 0.19 to *r* = 0.29. Credé and Phillips (2011) reported correlations between scales of the MSLQ and GPA, with the highest correlations for the scales effort regulation (*r* = 0.16), time and study environment (*r* = 0.17), and metacognitive self-regulation (*r* = 0.17). Importantly, the meta-analysis by Richardson et al. (2012) also addressed the key issue whether learning strategies can explain incremental variance even when controlling for the well-established predictor general cognitive ability. They found that effort regulation explained incremental variance over admission tests.

Given the above described benefit of females in educational achievement (e.g., Conger and Long, 2010) and disparities in the prediction of male and female academic success (e.g., Mellon et al., 1980), it is of special interest to also address gender differences in the prediction of AP. For the two sets of predictors that were of interest in the present study, general cognitive ability and learning strategies, previous evidence on gender differences is ambiguous.

When it comes to general cognitive ability, most previous studies did not report overall gender differences (for an overview see Halpern et al., 2011). However both sexes have their strengths and weaknesses in different tasks, such as a male advantage in different types of visual–spatial abilities and a female advantage in different memory tasks (e.g., Halpern and LaMay, 2000).

Differences in the predictability of AP by general cognitive ability were investigated less frequently. Gender differences were not found at school age (Freudenthaler et al., 2008; Steinmayr and Spinath, 2008). However, a well documented gender difference, known as the “female underprediction effect” (FUE), emerged when comparing the predicticted to the actual AP: standardized tests of cognitive ability usually overpredict men’s and underpredict women’s academic achievement (Kling et al., 2012), a finding that illustrates the need for testing gender-specific prediction models and the search for predictors of AP beyond general cognitive ability.

With respect to the predictor learning strategies, many studies found that female students reported more learning behavior than their male colleagues (e.g., Schiefele et al., 2003; Kesici et al., 2009; Virtanen and Nevgi, 2010; Marrs and Sigler, 2012; but see Richardson, 1993; Wilson et al., 1996; Chamorro-Premuzic and Furnham, 2009). Interestingly, Griffin et al. (2012) found that a significant relationship between gender and AP disappeared when controlling for individual differences in learning strategies, a finding that further highlights the importance of addressing gender differences in the prediction of AP by means of learning strategies.

Evidence for gender differences in the prediction of AP by learning strategies is scarce. Recently, Rosander and Bäckström (2012) explored the incremental contribution of learning approaches over general cognitive ability in school pupils while considering gender differences. Regression analyses showed that three different learning approaches explained incremental variance in girls’ AP but only one of them accounted for additional variance in boy’s AP.

To summarize, there is a large body of evidence pointing to the important role of general cognitive ability (e.g., Kuncel et al., 2004; Rohde and Thompson, 2007) and growing evidence for the importance of learning strategies (e.g., Credé and Phillips, 2011; Richardson et al., 2012) in predicting college academic achievement. However, more research is needed regarding the incremental contribution of specific learning strategies above general cognitive ability as predictor of AP. Additionally, as far as we know, prior research failed to examine if there are gender differences in the relative importance of this set of predictors for AP. Moreover, a further methodological weakness in prior learning research was the predominant application of univariate statistics. In contrast, the present study relied on latent multi-group modeling in order to test the gender differences in the incremental validity of learning strategies over general cognitive ability in the prediction of AP in university students.

Based on previous findings, we expected the following pattern of results: no gender differences in general cognitive ability (Halpern et al., 2011; Hypothesis 1), but gender differences in terms of learning strategies, that is, a more frequent use of these strategies in women (Schiefele et al., 2003; Kesici et al., 2009; Virtanen and Nevgi, 2010; Marrs and Sigler, 2012; Hypothesis 2). The literature review also lead us to expect positive correlations between general cognitive ability and AP (e.g., Lounsbury et al., 2003; Kuncel et al., 2004; Chamorro-Premuzic et al., 2006; Richardson et al., 2012; Hypothesis 3). Furthermore, learning strategies might be positively related to AP (Schiefele et al., 2003; Credé and Phillips, 2011; Griffin et al., 2012; Richardson et al., 2012; Hypothesis 4) and may explain incremental variance above general cognitive ability (Richardson et al., 2012; Hypotheses 5). Finally, we tested whether the predictability of AP by means of general cognitive ability and learning strategies varied as a function of gender.

## Materials and Methods

### Sample and Procedure

The sample investigated in this study was part of the German longitudinal Study on Individual and Organizational Influences on Study Performance in Teacher Education (SioS; Kaub et al., 2012; Reichl et al., 2014; Ruffing et al., 2015) at Saarland University. The project aimed at investigating professional competencies in teacher education by means of a longitudinal 6-years assessment. Students enrolled in the university teacher education program were tested on a large test battery including organizational, personal, and achievement characteristics. The study was approved by the ethics committee of the German Psychological Association. Participants were recruited by email or in a basic lecture. The present investigation was based on data from 461 students pursuing a teaching degree (mean age = 21.2 years, SD = 3.17; age range = 17–44 years; 67% female). The gender distribution in the present sample was representative for the population of teacher students. Participants provided informed written consent including the permission to access their academic grades in the university database.

### Measures

#### General Cognitive Ability

We used the short version of a well-established German test for general cognitive ability, Horn’s performance test system (LPS; Horn, 1983). This short version consisted of eight timed subtests with 40–65 items, assessing verbal (verbal comprehension, word fluency, word comprehension), spatial (spatial visualization), and reasoning abilities (reasoning) as well as perceptual speed (speed, number facility). For instance, participants were instructed to identify words in the verbal comprehension task and to count visible and hidden surfaces in three-dimensional figures in the spatial visualization task. Participants completed this paper–pencil test in about 30 min. Item responses were dichotomously scored and the total test score was the average of correctly solved items across subtests. The LPS is a standardized intelligence scale, which has shown good reliability estimates and has been validated against the IST (Intelligence Structure Test) and grades (Horn, 1983).

#### Learning Strategies

The Inventory for the Measurement of Learning Strategies in Academic Studies (LIST; Wild and Schiefele, 1994) was used to measure student’s self-reported use of learning strategies. This German paper–pencil inventory includes 77 items and measures the scales *Effort, Attention, Time management, Learning environment, Learning with fellow students* (resource-management strategies), *Organization, Relationships, Critical evaluation, Rehearsal* (cognitive strategies) as well as *meta-cognition.* Items were rated on a 5-point Likert scale ranging from 1 (strongly disagree) to 5 (strongly agree). Evidence for reliability and validity was presented by Boerner et al. (2005). In our study, we calculated mean values for each of the scales and their reliability coefficients (Cronbach’s α) ranged from 0.68 to 0.93 (see **Table 1**). The number of items included in each subscale is displayed in **Table 2.** We confirmed the dimensional structure using maximum likelihood confirmatory factor analysis by using items as well as item parcels for larger scales (χ^2^ = 1667.81, df = 724, *p* < 0.01, χ^2^/df = 2.30, CFI = 0.90, RMSEA = 0.05, SRMR = 0.06).

**Table 1:** Means (M), standard deviations (SDs), reliability coefficients, and correlations between AP, general cognitive ability and learning strategies.

Variables	*M*	SD	α	1	2	3	4	5	6	7	8	9	10	11	12
(1) Grade average	3.44	0.78													
(2) General cognitive ability	26.77	2.79	>0.77	0.23**											
(3) Effort	3.64	0.61	0.80	0.26**	0.05										
(4) Attention	3.25	0.83	0.93	0.25**	0.12*	0.51**									
(5) Organization	3.73	0.69	0.83	0.09	-0.01	0.36**	0.23**								
(6) Relationships	3.47	0.63	0.83	0.09	0.06	0.15**	0.10*	0.18**							
(7) Rehearsal	3.39	0.73	0.80	0.01	-0.12*	0.36**	0.09	0.39**	-0.05						
(8) Critical evaluation	2.89	0.70	0.86	0.02	-0.04	0.15**	0.17**	0.07	0.59**	0.12**					
(9) Time management	3.01	0.93	0.83	0.07	-0.04	0.42**	0.21**	0.28**	-0.01	0.35**	-0.02				
(10) Learning environment	3.79	0.65	0.77	0.11*	-0.06	0.51**	0.37**	0.38**	0.13**	0.34**	0.05	0.39**			
(11) Learning with fellow students	3.22	0.78	0.87	-0.04	-0.09*	0.08	0.08	0.13**	0.22**	0.10*	0.16**	0.13**	0.08		
(12) Literature	3.64	0.81	0.82	0.08	-0.06	0.36**	0.22**	0.26**	0.37**	0.20**	0.39**	0.02	0.20**	0.16**	
(13) Meta-cognition	3.61	0.47	0.68	0.09	-0.04	0.56**	0.38**	0.49**	0.35**	0.44**	0.30**	0.41**	0.40**	0.31**	0.39**

N = 461. The scales for the learning strategies ranged from 1 to 5 with five indicating more frequent application of the strategy. The scale for the grades ranged from 1 (insufficient) to 5 (very good). All internal consistencies refer to Cronbach’s α, except for the LPS. Guttman split-half coefficients for the subtests of the LPS revealed reliability coefficients ranging from *r* = 0.777 -*r* = 0.96. Correlations refer to bivariate correlations on the manifest level. *p < 0.05, **p < 0.01 (two-tailed).

**Table 2:** Means (M) and standard deviations (SDs) of the learning strategy scales as a function of gender and tests for gender differences (*t*-tests).

	Number of items	Female		Male		*T*-test for gender difference	Effect size
		*M*	SD	*M*	SD	*p*	*d*
(1) Effort	8	3.72	0.58	3.47	0.63	0.000*	0.42
(2) Attention	6	3.28	0.84	3.18	0.82	0.244	0.12
(3) Organization	8	3.89	0.62	3.41	0.72	0.000*	0.74
(4) Relationships	8	3.41	0.65	3.59	0.57	0.003*	-0.29
(5) Rehearsal	7	3.56	0.69	3.04	0.67	0.000*	0.76
(6) Critical evaluation	8	2.81	0.70	3.06	0.68	0.000*	-0.36
(7) Time management	4	3.10	0.95	2.83	0.87	0.004*	0.29
(8) Learning environment	6	3.85	0.66	3.68	0.61	0.008	0.26
(9) Learning with fellow students	7	3.22	0.78	3.22	0.77	0.975	0.00
(10) Literature	4	3.72	0.81	3.50	0.79	0.006	0.27
(11) Meta-cognition	11	3.66	0.48	3.49	0.43	0.000*	0.37

N = 461. The scales for the learning strategies ranged from 1 to 5 with five indicating more frequent application of the strategy. *p < 0.005 (Bonferroni-correction).

#### Academic Performance

Academic performance was measured by means of the participants’ grades in educational science. This included two written exams testing knowledge in the domains of “teaching and learning” and “personality development and education.” These exams are core components of the basic curriculum in teacher education and are comparable across all teacher students. Grades ranged from 1 (very good) to 5 (failed), and were recoded in order to facilitate the interpretation of the present findings (i.e., after recoding, higher values characterized higher achievement).

### Data Screening and Analysis

For the statistical analysis, we used the software packages SPSS 20 and Mplus 6 (Muthén and Muthén, 1998-2011). Prior to analysis, we carefully checked the data for missing values. The percentage of missing data was below 5% for all items and can therefore be considered negligible (Kline, 2011). Little MCAR test (missing completely at random; Little and Rubin, 2002), applied to compare observed variable means with the expected population means, indicated that missing data in the LIST occurred randomly (*p* > 0.05). We therefore imputed missing data in the LIST with the EM (expectation–maximization) algorithm.

To test our diverse set of hypothesis we combined different analytic methods. In a first step, we performed descriptive, reliability, and correlation analyses to describe the underlying sample as well as to check for violations of prerequisites and irregularities in the data set. Secondly, by applying *t*-tests and a Mann–Whitney *U* test we tested for gender differences in our set of variables.

**FIGURE 1:**
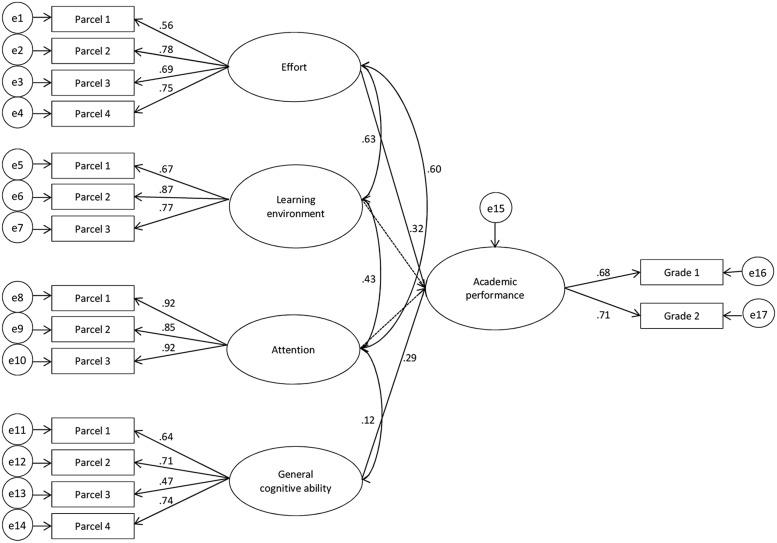
**Latent variable model for the structural model.** Grade1 = grade for the exam on “teaching and learning”, Grade2 = grade for the exam on “personality development and education.” Parcel 1–4: two-item parcels for each factor. e1–e17: error terms of the parcels and grades. Dashed lines indicate paths with insignificant coefficients.

In order to test the predictive validity of learning strategies over general cognitive ability for academic success in university students, we then conducted structural equation modeling (SEM). Applying this latent variable approach allowed us to consider complex relationships between our set of variables with the advantage of controlling measurement errors as well as examining intercorrelations between variables simultaneously (Geiser, 2010). Specifically, we investigated the unique contribution of the non-cognitive variable learning strategies to AP while controlling for the influence and any intercorrelation with general cognitive ability. Therefore, we specified a recursive model including four independent variables (general cognitive ability and the three learning strategies that were significantly correlated with AP, namely effort, attention, and learning environment), and the dependent variable (academic success; see **Figure 1**). Correlations between latent predictors were also allowed if their correlations reached significance on the manifest level. In this full or free model, we applied no restrictions and all paths were freely estimated. We subsequently fixed non-significant parameters to zero to specify a nested more parsimonious model. We used a chi-square difference test statistic to test if this model fitted the data equally well.

**FIGURE 2:**
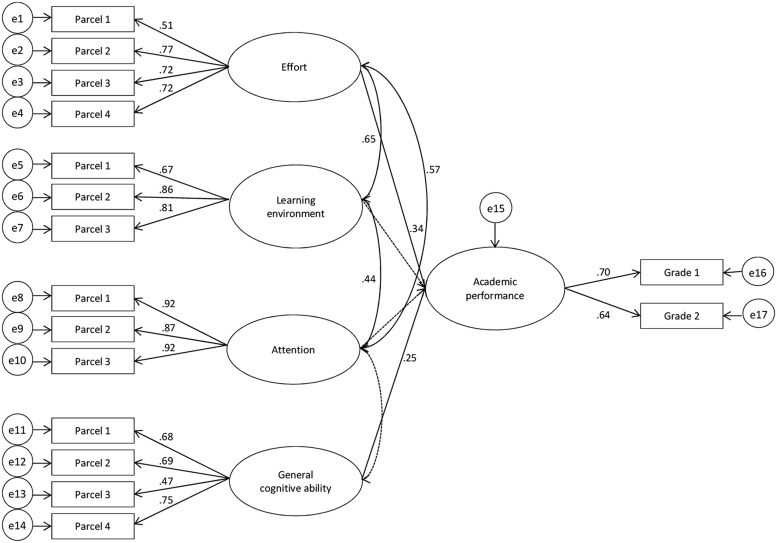
**Latent variable model for the prediction of female academic achievement**. Grade1 = grade for the exam on “teaching and learning,” Grade2 = grade for the exam on “personality development and education.” Parcel 1–4: two-item parcels for each factor. e1–e17: error terms of the parcels and grades. Dashed lines indicate paths with insignificant coefficients. Paths from learning environment and attention to academic performance (AP) have been fixed to zero.

**FIGURE 3:**
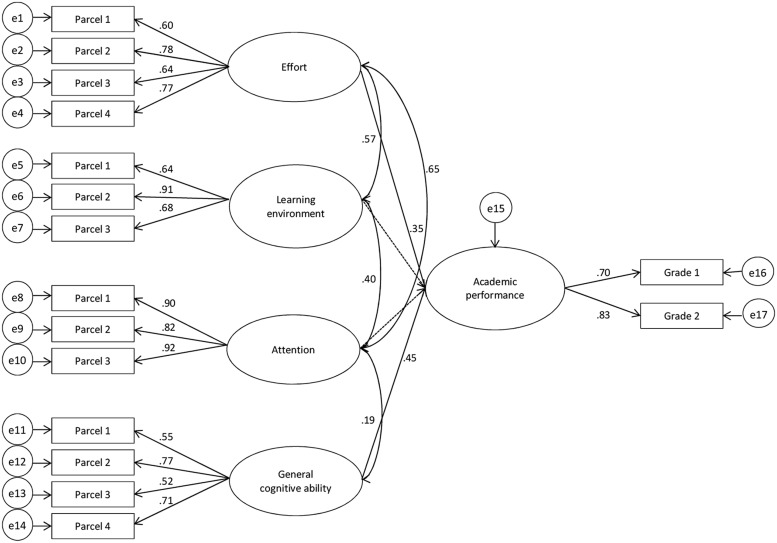
**Latent variable model for the prediction of male academic achievement.** Parcel 1–4: two-item parcels for each factor. Grade1 = grade for the exam on “teaching and learning,” Grade2 = grade for the exam on “personality development and education”. e1–e17: error terms of the parcels and grades. Paths from learning environment and attention to AP have been fixed to zero.

In a final step, we used multi-group analysis to investigate gender differences in the prediction of academic success. We examined invariance in different parameters of the measurement and structural model of female (**Figure 2**) and male (**Figure 3**) AP. We tested for gender differences through gender-specific differences in significance or a significant decline in model fit due to the restriction of parameters. This decline in model fit was identified by chi-square difference statistic, whereby a non-significant result indicated invariance in tested parameters.

For the invariance testing we hierarchically constrained different model parameters. In the configural model we set no between-group constraints. We then successively imposed equality constraints on factor loadings to initially test for measurement invariance. Afterward, we successively constrained covariances and finally regression weights in the structural model. Comparing chi-square difference statistics allowed us to test for gender-specific differences in measurement models, covariances, or path coefficients.

Evaluation of the SEM model fit followed suggestions from Kline (2005). Therefore, fit-indices reported in this study include the model chi-square, the Steiger–Lind root mean square error of approximation (RMSEA), the Bentler comparative fit index (CFI), and the standardized root mean square residual (SRMR). Given that the power of the chi-square test is sensitive to the sample size and the size of correlations (Kline, 2005), we also reported the relative chi-square (CMIN/DF). This index should not exceed a value of three (Schermelleh-Engel et al., 2003). For the RMSEA values should not be higher than 0.06 and for the SRMR values lower than 0.08 are desirable, and the CFI should at least reach a value of 0.95 (Hu and Bentler, 1999).

## Results

### Descriptive Statistics

Means, standard deviations, internal consistencies, and zero-order correlations for grade average, general cognitive ability, and learning strategies are shown in **Table 1**. The reliability coefficients of most scales exceeded α > 0.80; only the scale meta-cognition fell below the critical value of α > 0.70 (cf. Wild and Schiefele, 1994).

### Gender Differences in Predictors and Criterion

*T*-tests revealed gender differences for all learning strategies except for learning with fellow students, literature, learning environment, and attention. Male students more often relied on relationships and critical evaluation, whereas female students used all remaining strategies more often. Gender differences in organization and rehearsal were medium sized in Cohen’s (1988) terms and small for effort, relationships, time management, meta-cognition, and critical evaluation. In terms of general cognitive ability we found no significant differences between females (*M* = 26.66, SD = 2.71) and males (*M* = 26.99, SD = 2.94; *t*(459) = -1.19, *p* = 0.23). Regarding academic success, a Mann–Whitney *U* test also revealed no significant gender difference *(Z* = -1.02, *p* > 0.05).

### Correlations between Academic Achievement and Predictors

As expected, general cognitive ability was positively correlated with academic grades (*r* = 0.23, *p* < 0.01; see **Table 1**). For the learning strategies, we found that effort (*r* = 0.26, *p* < 0.01), attention (*r* = 0.25, *p* < 0.01), and learning environment (*r* = 0.11, *p* < 0.05) were positively related to academic achievement. Thus, higher values of general cognitive ability and a more frequent application of learning strategies were associated with better AP. However, the correlations were small in Cohen’s (1988) terms. The remaining learning strategies organization, relationships, rehearsal, critical evaluation, time management, learning with fellow students, literature, and meta-cognition were not significantly related to academic achievement. Intercorrelations between general cognitive ability and learning strategies were generally small (all *r’s* < 0.12), and only the correlations between general cognitive ability and attention (*r* = 0.12, *p* < 0.05), rehearsal (*r* = -0.12, *p* < 0.05), and learning with fellow students (*r* = -0.09, *p* < 0.05) reached significance. Higher general cognitive ability was associated with higher levels of attention and lower levels of rehearsal and learning with fellow students. In contrast, most of the learning strategies were significantly intercorrelated. For instance, large effects were observed for effort and attention (*r* = 0.51, *p* < 0.01), medium effects for organization and effort (*r* = 0.36 *p* < 0.01) and small effects for literature and rehearsal (*r* = 0.20, *p* < 0.01; for a detailed overview see **Table 1**).

### Structural Model

The endogenous variable AP in the structural model was defined by academic grades, whereas the exogenous variables were defined by the cognitive predictor general cognitive ability as well as the non-cognitive predictor learning strategies. By simultaneously examining the set of exogenous variables we aimed at testing for the unique contribution of learning strategies for AP. For each of the latent predictors, we parceled items according to the item-to-construct balance technique (Little et al., 2002). The full model, in which no restrictions were applied, provided a good fit to the data (χ^2^ = 134.14, df = 96, *p* < 0.05, χ^2^/df = 1.40, CFI = 0.99, RMSEA = 0.03, SRMR = 0.04), but only the paths from general cognitive ability (β = 0.29; *p* < 0.01) and effort (β = 0.32; *p* < 0.01) to AP reached statistical significance, while attention and learning environment had no significant impact (both *p*-values > 0.05, see **Figure 1**). The total variance explained was 23% with effort explaining an additional 10% over general cognitive ability. Given that more parsimonious models have the theoretical and practical advantage that they are easier to replicate and explain (cf. Bentler and Mooijaart, 1989), we compared results of this full model to a nested model with all non-significant paths fixed to zero (χ^2^ = 138.51, df = 98, *p* < 0.01, χ^2^/df = 1.41, CFI = 0.99, RMSEA = 0.03, SRMR = 0.04). Chi-square difference statistics showed no significant difference, indicating that the most parsimonious model did not fit the data significantly worse than the full model (χ^2^_diff_ = 4.37, df_diff_ = 2, *p* = 0.11).

In order to compare estimates across males and females, we specified this model as a multi-group model (M_1_, **Figures 2** and **3**). The total variance explained in academic success was 18% for female students and 32% for male students. Effort explained 12% of the variance in females and 12% in males over general cognitive ability. Most importantly, the strongest predictor for female academic success was effort (β = 0.34, *p* < 0.01), followed by general cognitive ability (β = 0.25, *p* < 0.01). In contrast, the strongest predictor for male academic success was general cognitive ability (β = 0.45, *p* < 0.01) followed by effort (β = 0.35, *p* < 0.01). This model yielded a good fit to the empirical data (χ^2^ = 288.15, df = 196, *p* < 0.01, χ^2^/df = 1.47, CFI = 0.97, RMSEA = 0.05, SRMR = 0.05). We then performed a series of nested model comparisons to test for gender invariance. In order to illustrate this procedure, the fit indices, the order of compared models as well as χ^2^-difference statistics are presented in **Table 3**.

**Table 3:** Model fit indices and χ^2^-difference test of the nested models predicting male and female AP.

Model	Model description	χ^2^	df	χ^2^/df	CFI	RMSEA	SRMR	Δχ^2^	Δdf	*p*
M_1_	Configural model	288.15	196	1.47	0.97	0.05	0.05			
M_2_	M_1_ + factor loadings constrained equal across gender	302.68	207	1.46	0.97	0.05	0.06	14.53	11	0.21
M_3_	M_2_ + covariances between the latent factors constrained equal across gender	306.62	210	1.46	0.97	0.05	0.07	3.94	3	0.27
M_4_	M_3_ + paths in the structural model constrained equal across gender	308.68	212	1.46	0.97	0.04	0.07	2.07	2	0.36

Constraining the factor loadings corroborated metric invariance (M_1_ vs. M_2_: χ_diff_^2^ = 14.53, df_diff_ = 11, *p* = 0.21). Thus, the measurement models could be assumed equal for male and female students. As the correlation coefficient between attention and general cognitive ability was significant for male but not for female students, we decided not to set them equal in the following steps. To test whether the prediction model differed between male and female students, we constrained correlations (M_2_ vs. M_3_: χ_diff_^2^ = 3.94, df_diff_ = 3, *p* = 0.27) and path coefficients (M_3_ vs. M_4_: χ_diff_^2^ = 2.07, df_diff_ = 2, *p* = 0.36) in a stepwise procedure to be equal across groups. Given the lack of statistical significance regarding the chi-square difference, it could be assumed that these models did not fit significantly worse than the unconstrained model, suggesting that there was no gender difference in the prediction models. Thus, it was concluded that the predictive value of general cognitive ability and effort is not different for male and female AP.

## Discussion

### General Discussion

The prediction of AP is a major goal in psychological and educational research. Therefore, the first purpose of this study was to investigate the relative contribution of learning strategies to AP. It has been considered a promising predictor in previous research, especially when compared with the relatively stable and established construct of general cognitive ability. Second, to our knowledge this study is the first one to apply a multi-group modeling approach to examine gender differences with regard to the role of learning strategies and general cognitive ability for AP.

We replicated previous findings by showing that there were no gender differences in general cognitive ability (Halpern et al., 2011). Furthermore, we reported additional evidence for Marrs and Sigler’s (2012) finding that there are gender differences in the application of many learning strategies (see also Schiefele et al., 2003; Kesici et al., 2009; Virtanen and Nevgi, 2010). Apart from critical evaluation and relationships (used more often by male students) as well as literature, learning environment, learning with fellow students and attention (no gender differences), female students applied all remaining learning strategies more frequently. Interestingly, these specific strategies loaded on a factor learning discipline, which was closely related to conscientiousness in prior studies (Blickle, 1996). This finding was also consistent with previous results showing that woman scored higher on conscientiousness (e.g., Schmitt et al., 2008), a trait that has been linked to the FUE (Kling et al., 2012). The fact that female students generally used learning strategies more often may also indicate a different attitude toward academic studies, which is compatible with the results of the National Freshman Attitudes Report, showing that male students’ attitudes indicated lower academic engagement (Noel-Levitz, 2012). However, the gender gap in academic achievement (e.g., Conger and Long, 2010) was not replicated in the current investigation.

The examination of the association between learning strategies, general cognitive ability, and academic achievement revealed that consistent with the literature, general cognitive ability was associated with academic success (e.g., Lounsbury et al., 2003; Chamorro-Premuzic et al., 2006; Richardson et al., 2012). The fact that these associations were rather small is also consistent with previous findings (e.g., Richardson et al., 2012) and may reflect a restriction of variance in the sample investigated in the present study. In terms of learning strategies, a more differentiated picture emerged. The correlation between AP and the two strategies effort and attention surpassed the correlation between general cognitive ability and AP, highlighting the importance of learning and study skills as promising factors in the academic context (Credé and Kuncel, 2008). All strategies significantly related to AP belonged to the category of resource-related strategies. Such strategies serve to focus resources toward the actual learning process and to screen it from external influences, also characterized as self-management activities to organize learning activities (Wild and Schiefele, 1994). Effort, which was the most powerful strategy, is for example determined by working hard on weekends/late in the evening or more than study colleagues. Attention, in contrast to other learning strategies, refers to the indirect consequences of paying insufficient attention (Wild and Schiefele, 1994). The essential role of resource-management strategies in grade-related academic achievement tied in with previous research (e.g., Boerner et al., 2005) and especially the dominant role of effort also confirmed previous research (e.g., Schiefele et al., 2003).

The lack of significant associations between AP and other learning strategies may also be related to a discrepancy between the students’ self-reported and actual study behaviors (Jamieson-Noel and Winne, 2003). According to Pokay and Blumenfeld (1990, p. 47–48), self-reported strategy use assesses “only what students think they are doing and does not address the accuracy of these perceptions.” Thus, reporting self-management strategies may require less self-reflection than monitoring cognitive and metacognitive processes. Moreover, it has been argued that the low correlations between some of the strategies may be related to the way they were operationalized, particularly in terms of “grain size”: Jamieson-Noel and Winne (2003) assumed that students may apply diverse individual strategies while learning. However, if these are aggregated to larger clusters of study activities, such as strategies, their internal consistencies may be hampered which could reduce their impact in the prediction of criterion variables such as AP. Finally, a more practical concern implies that the learning environment at college may contribute to the more frequent use of less sophisticated learning strategies (cf. Wild, 1996). The reproduction of facts and knowledge required in many exams and classes may lead students to drop metacognitive strategies in order to save time and energy and focus on supporting information processing by optimizing learning–related resources instead.

Given that recent research intended to improve the prediction of AP by examining non-cognitive in addition to established cognitive predictors (Credé and Kuncel, 2008), learning strategies and general cognitive ability were examined in one structural equation model. The results regarding the incremental validity of learning strategies suggest that effort was the only learning strategy adding incremental variance (10%) which supports and extends previous findings (e.g., Richardson et al., 2012). In sum, these findings support Credé and Kuncel’s (2008) claim that study habits and skills may be among the most important non-cognitive predictors of AP in college students.

By using latent multi-group analyses we assessed gender differences in the prediction of AP. Thus, to our knowledge, ours is the first study overcoming previous methodological issues such as relying on univariate statistics alone to examine gender differences in learning behavior (Marrs and Sigler, 2012) and to test for gender differences in their predictability of academic success. The invariance testing revealed that the relative importance of effort and general cognitive ability was not significantly different between genders. The finding that the prediction model did not vary as a function of gender may suggest that although male students report using less learning strategies, their predictive value is not different from females. Thus, the same prediction model could be assumed for males and females and effort was an important incremental predictor beyond general cognitive ability regardless of gender. However, the differences in total variance explained (18% for females and 32% for males) and in the relative contribution of general cognitive ability to AP (regression weights of 0.45 in males and 0.25 in females) call for a further examination of gender differences in future studies.

### Limitations of the Current Study

Although our study overcame some of the methodological challenges of previous studies by applying a latent multi-group modeling approach and by assessing multiple, differentiated learning strategies, there are limitations that need to be acknowledged and discussed. As already mentioned, self-report questionnaires may not always be the most appropriate way to assess learning strategies. Other methods, such as think-aloud protocols might be interesting alternatives worth investigating (Pokay and Blumenfeld, 1990). Another criticism of self-report questionnaires is the predominant focus on quantitative instead of qualitative aspects of the strategy use. A further issue related to our learning strategy questionnaire is that its structure, including cognitive, metacognitive, and resource-management strategies, needs further confirmation from factor analytic studies.

Further difficulty always arising in samples of college students is the issue of pre-selection, especially when the focus is on performance-related variables, such as general cognitive ability and learning strategies. This variance restriction might underestimate true associations and therefore limit the generalizability of the present findings to other populations. Thus, future research might also consider other participants than college student samples.

Preceding results, such as the work of Pokay and Blumenfeld (1990), pointed to changes in success-related learning behavior over time and demonstrated the need for further longitudinal research investigating gender differences in learning strategies controlling for general cognitive ability over the course of college education. A further limitation of the current study was the primary focus on grades as criterion variable as other constructs are also important academic outcome variables (e.g., retention). Thus, for future studies it might be an important issue to broaden the criteria for the assessment of academic achievement (cf. Kunina et al., 2007).

## Conclusion

In sum, the present study yielded new findings regarding the differential contributions of learning strategies to AP. Irrespective of gender, effort appeared to be the one learning strategy that adds incremental variance over cognitive ability. Importantly, we integrated two central issues of the research on AP, namely the search for its prerequisites and research on group differences such as gender-related differences in academic context (cf. Spinath, 2012).

Given that the overall aim of educational research and interventions is to maximize the learner’s academic achievement according to their individual potential (cf. Spinath, 2012), our study has practical implications for the way students organize their learning behavior and for the way educational institutions support and counsel their students. Considering that there was evidence pointing to the effectiveness of interventions designed to improve learning behaviors (e.g., McKeachie et al., 1985; Hattie et al., 1996; Schatteman et al., 1997; Hofer and Yu, 2003) and that our findings pointed to the dominating role of resource-related learning strategies, interventions teaching and optimizing these learning strategies may be designed and implemented. Thus, given that learning strategies can be changed and modified, it seems worth considering them in the prediction of AP. Especially in the education of prospective teachers, learning strategies should be forstered because “teachers should be learners, not only in developing their practice, but also in modeling for their learners the process of continual learning” (Hagger et al., 2008, p. 160).

Regarding the investigated gender differences in learning strategy use, we can conclude that there are differences in preferred learning strategies between males and females which teachers should acknowlege, for example in order to reach students through specific instructions and presentation styles. However, students should also be aware of their most suitable and promising strategies and might benefit from each other, for instance through mixed learning groups. Moreover, our study indicated that the incremental assessment of learning strategies in college admission decisions might increase the predictability of academic success beyond the most common predictor of intelligence regardless of gender. However, more research might be nesseseray to further investigate the relatively large differences in explained variance between male and female AP. Finally, our study supports future research searching for alternative, incremental and non-cognitive predictors of AP and extends the understanding of the prerequisites of academic success.

## Conflict of Interest Statement

The authors declare that the research was conducted in the absence of any commercial or financial relationships that could be construed as a potential conflict of interest.
